# Validation of reference genes for quantitative RT-PCR normalization in *Suaeda aralocaspica*, an annual halophyte with heteromorphism and C4 pathway without Kranz anatomy

**DOI:** 10.7717/peerj.1697

**Published:** 2016-02-11

**Authors:** Jing Cao, Lu Wang, Haiyan Lan

**Affiliations:** Xinjiang Key Laboratory of Biological Resources and Genetic Engineering, College of Life Science and Technology, Xinjiang University, Urumqi, China

**Keywords:** Reference gene, Quantitative RT-PCR, *Suaeda aralocaspica*, Abiotic stress, Normalization, Heteromorphism

## Abstract

Reverse transcription quantitative real-time polymerase chain reaction (qRT-PCR) is a powerful analytical technique for the measurement of gene expression, which depends on the stability of the reference gene used for data normalization. *Suaeda aralocaspica*, an annual halophyte with heteromorphic seeds and possessing C4 photosynthesis pathway without Kranz anatomy, is an ideal plant species to identify stress tolerance-related genes and compare relative expression at transcriptional level. So far, no molecular information is available for this species. In the present study, six traditionally used reference genes were selected and their expression stability in two types of seeds of *S. aralocaspica* under different experimental conditions was evaluated. Three analytical programs, geNorm, NormFinder and BestKeeper, were used to assess and rank the stability of reference gene expression. Results revealed that although some reference genes may display different transcriptional profiles between the two types of seeds, *β*-TUB and *GAPDH* appeared to be the most suitable references under different developmental stages and tissues. *GAPDH* was the appropriate reference gene under different germination time points and salt stress conditions, and *ACTIN* was suitable for various abiotic stress treatments for the two types of seeds. For all the sample pools, *β*-TUB served as the most stable reference gene, whereas *18S rRNA* and *28S rRNA* performed poorly and presented as the least stable genes in our study. *UBQ* seemed to be unsuitable as internal control under different salt treatments. In addition, the expression of a photosynthesis-related gene (*PPDK*) of C4 pathway and a salt tolerance-related gene (*SAT*) of *S. aralocaspica* were used to validate the best performance reference genes. This is the first systematic comparison of reference gene selection for qRT-PCR work in *S. aralocaspica* and these data will facilitate further studies on gene expression in this species and other euhalophytes.

## Introduction

*Suaeda aralocaspica*, the only species of *Borszczowia* section of *Suaeda* genus in the Amaranthaceae, is a monoecious annual halophyte distributed in the saline-alkaline sandy soils in the southern margin of Junggar Basin in China (Li, 1979; Mao, 1994). It not only produces heteromorphic seeds with different forms and germination characteristics on a single plant (Wang et al., 2008), but also possesses unique C4 photosynthesis pathway without Kranz anatomy (Voznesenskaya et al., 2001), which allows the photosynthesis to perform within a single elongated chlorenchyma cell (Voznesenskaya, Franceschi & Edwards, 2004). More interestingly, the seedling of *S. aralocaspica* showed delayed developmental phenomenon to get through the harsh natural conditions (e.g., high salinity, remarkable daily temperature variation, strong light, etc.) in early spring (J Cao et al., 2016, unpublished data). All of these characteristics endow *S. aralocaspica* with remarkably abiotic tolerance and high efficiency of photosynthesis; however, how can this species adapt to such a harsh environment, e.g., the molecular mechanism of the stress tolerance, is still poorly-understood.

To provide insight into these complex stress-responsive regulatory networks and identify stress-responded genes, high throughput gene expression analysis should be considered (Delporte et al., 2015). Reverse transcription quantitative real-time PCR (qRT-PCR) is one of the most widely used technologies to validate small sets of gene expression or the whole-genome microarray data, due to its high sensitivity and accuracy, good reproducibility, as well as the broad dynamic range for a limited number of target genes (Higuchi et al., 1993; Heid et al., 1996; Huggett et al., 2005). However, the accuracy of qRT-PCR remains influenced by many experimental variations (Bustin, 2002; Ginzinger, 2002; Udvardi, Czechowski & Scheible, 2008; Bustin et al., 2009); among them, selection of a constant expression reference gene for normalization is crucial for the acquisition of biological meaningful data (Thellin et al., 1999; Schmittgen & Zakrajsek, 2000). So far, the traditional reference genes involved in basic cellular maintenance such as 18S ribosomal RNA (*18S rRNA*), 28S ribosomal RNA (*28S rRNA*), cytoskeletal structural protein *β*-actin (*ACTIN*), *β*-tubulin (*β-TUB*), glyceraldehyde-3-phosphate dehydrogenase (*GAPDH*) and ubiquitin protein (*UBQ*) were largely used for quantification of mRNA expression in model plants, animals and human beings (Remans et al., 2008; Shen et al., 2010; Ponton et al., 2011; Du et al., 2013). However, several reports demonstrate that the copy of transcript of these genes does not always maintain stable under different experimental conditions (Bas et al., 2004; Czechowski et al., 2005; Exposito-Rodriguez et al., 2008; Tong et al., 2009; Die et al., 2010; Zhu et al., 2013). If the chosen reference gene exhibits large expression fluctuation, the normalization will lead to inaccurate gene expression profile of target genes and inappropriate interpretation of biological data (Czechowski et al., 2005; Tong et al., 2009; Bustin et al., 2009; Artico et al., 2010). Therefore, it is essential to choose suitable reference genes for each species and ensure their stability under the specifically experimental conditions (Li et al., 2015).

With the growing awareness of significance of suitable reference gene, several statistical algorithms, such as geNorm (Vandesompele et al., 2002), NormFinder (Andersen, Jensen & Orntoft, 2004), BestKeeper (Pfaffl, Prgomet & Neuvians, 2004), qBasePlus (Hellemans et al., 2007) and RefFinder (Xie et al., 2012) have been well developed to validate the most stable reference gene(s) from candidates under a given set of experimental conditions. Recently, an increasing number of reports have focused on systematic validation of stable reference genes in model plants (Remans et al., 2008; Schmidt & Delaney, 2010; Lilly et al., 2011), important crop species (Kim et al., 2003; Jain et al., 2006; Jian et al., 2008; Hu et al., 2009; Paolacci et al., 2009; Janská et al., 2013; Lin et al., 2014), vegetables (Nicot et al., 2005; Die et al., 2010; Gantasala et al., 2013; Tian et al., 2015) and fruits (Reid et al., 2006; Tong et al., 2009; Chen et al., 2011; Clancy et al., 2013; Chen et al., 2015). However, only few studies have been conducted to identify the suitable reference genes in desert species (Li et al., 2012; Shi et al., 2012; Zhu et al., 2013; Yan et al., 2014; Li et al., 2015). To our knowledge, no work has been reported to evaluate reference genes for an euhalophyte in desert. In the present study, we cloned and evaluated the stability of six commonly used reference genes (*18S rRNA*, *28S rRNA*, *ACTIN*, *TUB*, *GAPDH*, *UBQ*) based on their expression abundance in two types of plants derived from heteromorphic seeds of *S. aralocaspica*. Samples were collected from different tissues, developmental stages and various stress treatments. The best reference genes from the candidates were tested by normalizing expression of a C4 photosynthesis-related gene-pyruvate orthophosphate dikinase (*PPDK*) and a salinity tolerance-related gene-salt induced AAA-Type ATPase (*SAT*). qRT-PCR data were analyzed using three widely applied algorithms-geNorm, NormFinder and BestKeeper to determine sets of reference genes suitable for *S. aralocaspica* in different experimental conditions. Our work should facilitate future study on gene expression analysis in *S. aralocaspica* and other euhalophytes, which will then improve our understanding of the molecular mechanisms of desert plant adaptation to salt stress.

## Material and Methods

### Plant materials and treatments

Mature seeds of *Suaeda aralocaspica* were collected from Gurbantunggut Desert of Xinjiang Uyghur Autonomous Region of China (Wujiaqu 103 regiment, 44°29′821″N, 87°31′181″E) in October, 2014. Seeds were naturally air-dried indoor, then cleaned and sieved to remove the impurities, and stored at 4 °C in brown paper bag until use.

#### Seed germination

Four replicates with 45 seeds of each of the two morphs of *S. aralocaspica* were sown on two layers of moist filter paper in a 9-cm Petri dish and exposed to different stress conditions to evaluate the stability of the tested reference genes. 40 germinated (brown) or non-germinated (black) seeds of each sample were collected on day 1 and 7 after sowing, dry seed at day 0 was used as control. The stress treatments included salinity, drought, low temperature (conditions experienced by *S. aralocaspica* in its natural habitat), ABA and H_2_O_2_ (factors used in alteration of gene expression in plants). Filter paper was saturated with 6 mL of distilled water or the following aqueous solutions: 300 mM NaCl, 20% (w/v) polyethylene glycol (PEG) 6000, 1 µM ABA and 0.01% H_2_O_2_, respectively. All Petri dishes were kept in an illumination incubator (RXZ-5000C, Ningbo Jiangnan Instrument Factory, China) at constant 25 °C (or 10 °C in low temperature stress) and a photoperiod of 16 h light/8 h dark, the light intensity was 396 µmol/m^2^/s.

#### Seedling growth

*S. aralocaspica* seeds were sown in pots containing perlite: vermiculite (1:3) under a 16 h light/8 h dark photoperiod at a temperature regime of 24–30 °C, 10–20% relative humidity and a light source of 500–700 µmol/m^2^/s. Before sowing, the black seeds were stratified for 10 d according to Wang et al. (2008) to synchronize seed germination. Subsequently, the brown and black seeds were sown at the same time. Seedlings were cultivated with half-strength Hoagland solution (Arnon & Hoagland, 1940) containing 100, 300, 500 mM NaCl, half-strength Hoagland solution only was used as control. For salt treatment, only two cotyledons of the seedling were collected as sample in the presence of various salt concentrations on day 15 after seedling emergence. For different tissues, samples were collected from cotyledons, stem and root of seedling in the absence of salt on day 15 after emergence. For different developmental stages, samples were collected from the whole seedling grown in the absence of salt on day 3, 15, 30 and 60 after emergence. All samples were immediately frozen in liquid nitrogen on harvesting and stored at −80 °C prior to RNA extraction.

### RNA extraction and cDNA synthesis

Total RNA was extracted by using RNAprep Pure Plant Kit (Tiangen, Beijing, China) according to the manufacture’s instructions. The ratios of absorbance at 260 nm to that of 280 nm (260/280) or 230 nm (260/230) were used to assess the purity of RNA using a Nanodrop ND-1000 UV Spectrophotometer (Thermo Fisher Scientific, Waltham, MA, USA), only RNA samples with a 260/280 ratio between 1.9 and 2.1 and 260/230 ratio higher than 2.0 were used for subsequent analysis. RNA integrity was visualized via 1% (w/v) agarose gel electrophoresis with two clear bands of 28S/18S ribosomal RNA. Each reverse transcription reaction was performed with 1 µg of total RNA in a final volume of 20 µL by using M-MLV RTase cDNA Synthesis Kit (D6130, TaKaRa, Shiga, Japan) with 2.5 µM oligo(dT) and 5 µM random hexamer primer following the manufacturer’s instructions. cDNA was stored at −20 °C before proceeding to the next step.

#### Cloning the partial sequences of the candidate reference genes

Based on previously reported qRT-PCR reference gene in Arabidopsis, we selected six commonly used reference genes spanning a range of biological functions as candidates in *S. aralocaspica*: 18S ribosomal RNA (*18S*), 28S ribosomal RNA (*28S*), cytoskeletal structural protein *β*-actin (*ACTIN*), *β*-tubulin (*β-TUB*), glyceraldehyde-3-phosphate dehydrogenase (*GAPDH*) and ubiquitin protein (*UBQ*). According to the published sequences in Amaranthaceae or Caryophyllales, the partial sequences of the six candidate genes were obtained from homology-based cloning. The EST fragments of pyruvate orthophosphate dikinase gene (*PPDK*) and salt-induced AAA-Type ATPase gene (*SAT*), which were screened from our previously transcriptomic data of *S. aralocaspica* (J Cao et al., 2016, unpublished data), were used as the target genes for reference gene validation.

PCR reaction was performed using TaKaRa Taq™ (TaKaRa, Shiga, Japan) at the conditions as follows: 35 cycles with denaturation at 94 °C for 30 s, annealing at 55–65 °C (depending on melting temperature of each pair of gene-specific primers) for 30 s, and extension at 72 °C for 30 s; an initial denaturation step of 5 min at 94 °C and a final elongation step at 72 °C for 10 min were performed. Amplicons were purified using the TIANgen Midi Gel Purification Kit (Tiangen, China) and cloned into pMD™ 18-T vector (TaKaRa, Shiga, Japan) according to manufacturer’s instructions. Positive colonies containing recombinant plasmids were then sent to Beijing Genomics Institute (Beijing, China) for DNA sequencing. The resulting DNA sequence was used to query the appropriate databases using the BLAST algorithm of NCBI (Table S1).

### Specific primer design and quantitative real-time PCR

Based on the gene sequence obtained from homology-based cloning, all gene-specific primer sets for qRT-PCR were designed using DNAMAN 5.0 according to the following requirements: the amplicon size is from 100 to 300 bp and the Tm value is about 60 ± 5 °C (Table 1). Amplification efficiency was evaluated using a standard curve generated by qRT-PCR using a ten-fold dilution series over at least four dilution gradients with three replicates of each. Primer specificity was confirmed by gel electrophoresis and melting-curve analysis based on qRT-PCR performance.

**Table 1:** Gene-specific primers and amplicon characteristics of candidate reference genes and target genes used in qRT-PCR analysis.

Gene name	Length (bp)	Tm (°C)	Amplification efficiency (%)	*R*^2^	5′-sequence (forward primer)	3′-sequence (reverse primer)
*18S*	256	85.44	97.76	0.992	GCGGCTTAATTTGACTCAACACG	CCTGTTATTGCCTCAAACTTCC
*28S*	154	85.78	92.89	0.998	GCCGACCCTGATCTTCTGTGA	TACCCAAGTCAGACGAACGATT
*ACTIN*	149	82.27	93.34	0.996	CCAAAGGCCAACAGAGAGAAGAT	TGAGACACACCATCACCAGAAT
*β-TUB*	223	85.47	94.36	0.999	CCTTATTCCATTCCCCAGGCTTC	CATCTGCTCATCAACCTCCTTTGTGC
*GAPDH*	162	83.37	93.75	0.998	GTTTTCACTGACAAGGACAAGGCTGCTG	GGTGGTACAACTGGCATTGGAGAC
*UBQ*	127	81.39	93.18	0.999	GCTAAGATTCAAGACAAGGAGGGTATC	CCAGGTGGAGGGTTGATTCTTTCTG
*PPDK*	144	80.49	95.93	0.994	CTGTCCCAGGAGTCAAACAC	CACTGAACTAACTGCTTCCGA
*SAT*	168	88.35	97.12	0.994	GAGTATCTCCGAAGAGCCGAG	GCATCCTCACCATCCTTCTC

qRT-PCR reaction was performed using GoTaq^®^ qPCR Master Mix (Promega, Madison, WI, USA) in the GeneAmp^®^ 7500 Real-Time PCR System (ABI, Vernon, CA, USA). The reaction mixture consisted of 1 µL cDNA samples, 0.5 µL of each of the forward and reverse primers (10 µM), 10 µL GoTaq^®^ qPCR master mix and 8 µL nuclease-free water in a final volume of 20 µL. Four biological replicates with two technical replicates of each for all samples were applied. The qRT-PCR was performed as follows: 2 min initial denaturation at 95 °C, 40 cycles of 95 °C for 15 s and 60 °C for 1 min. The crossing cycle number (CT) of each reaction was automatically determined by the GeneAmp^®^ 7500 software with default parameters.

### Statistical analysis of gene expression stability

Three different types of Microsoft Excel-based software (geNorm, NormFinder and BestKeeper) were used to rank the stability of reference genes in all experiments. All three software packages were used according to the manufacturer’s procedures.

For geNorm, the raw CT value was imported into Microsoft Excel first, and transformed into relative expression level using the formula *Q* = 2^(minCT−sampleCT)^ (the maximum expression level of each gene was used as control and was assigned a value of 1) and then introduced into geNorm (version 3.5). The expression stability value (*M*) for each gene and the pairwise variation value (*V*) of the target gene with other genes were further calculated using geNorm algorithm. All of the tested genes were ranked according to their *M* values (the cutoff of *M* value was set as 1.5, the lower value suggests the more stable gene expression) and then the optimal number of reference genes for normalization was determined. NormFinder employed the same input file format as geNorm, while the BestKeeper analysis was based on the untransformed CT values.

### Normalization of the target genes

To assess the validity of the programs used in ranking the reference genes above, *PPDK* and *SAT* gene of *S. aralocaspica*, which were characterized in ‘Cloning the partial sequences of the candidate reference genes’ section, were used as the target genes. The expression level of these genes was quantified using the most stable reference genes determined by geNorm, NormFinder and BestKeeper. Samples were collected from seedlings or seeds in germination treated by 300 mM NaCl for 0, 1 and 7 d. The relative expression level of target gene can be quantified according to the mathematical model *R* = 2^−ΔΔCT^ (Shi & Chiang, 2005), where ΔΔCT = ΔCT_targetsample_ − ΔCT_controlsample_, ΔCT_sample_ = CT_testgene_ − CT_referencegene_. The final value of relative quantification was described as fold change of gene expression in the test sample compared to control. Data were expressed as mean ± SE of eight replicates for each sample.

## Results

### Verification of amplicon and primer specificity and amplification efficiency

**Figure 1:**
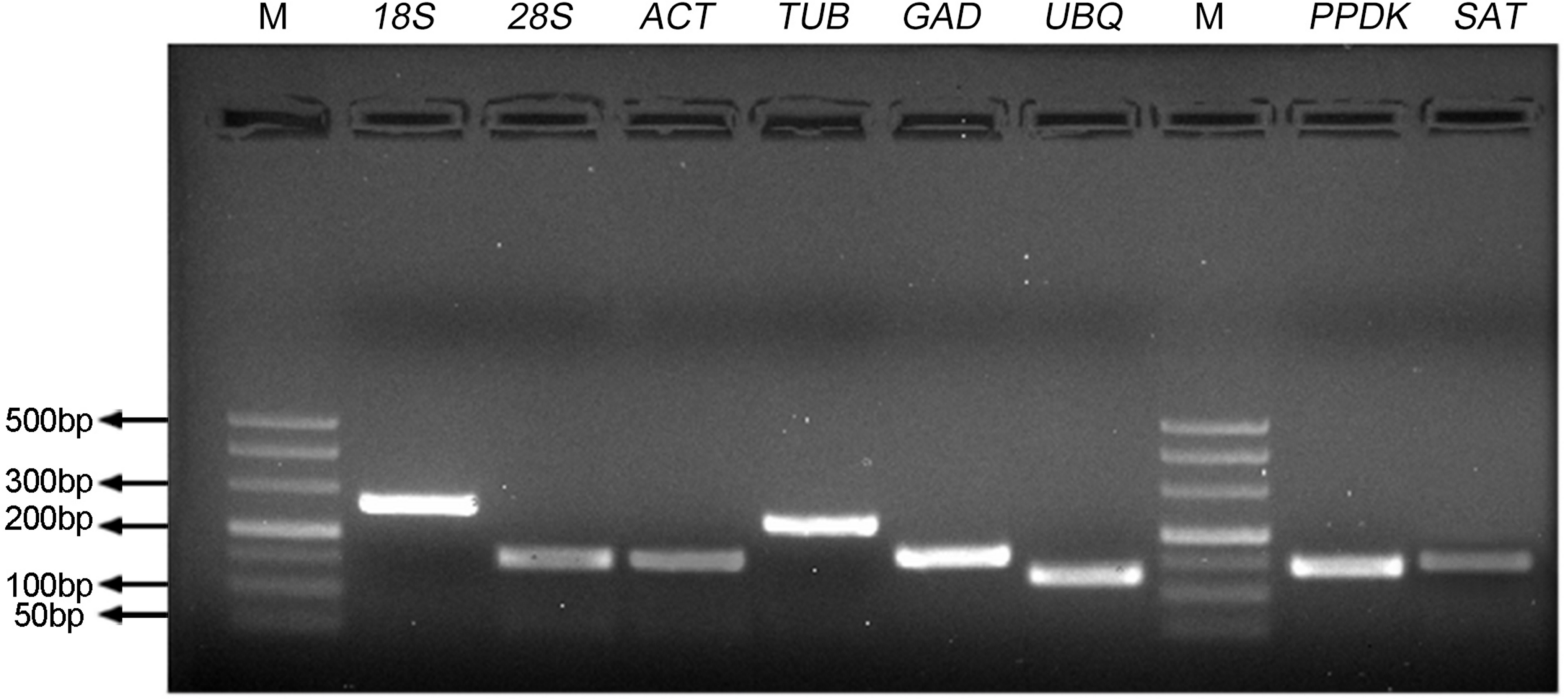
Specificity of primer pairs for qRT-PCR amplification. The mixture of equal amount of cDNAs from all tested samples was used as the template. 3.0% agarose gel electrophoresis visualized the specific amplification for each reference gene with the expected size. M stands for DNA size marker.

To identify the stability of reference genes for gene expression analysis of *S. aralocaspica,* the qRT-PCR assay was performed with the proper fragment of six candidate reference genes (*18S rRNA, 28S rRNA, ACTIN, β-TUB, GAPDH* and *UBQ*). Only a single band of each amplicon was generated from cDNA samples with the size from 127 bp to 256 bp (Fig. 1). They shared 84%–100% identity with the corresponding gene sequences from other plant species (Tabl S1). A single peak melting curve in qRT-PCR confirmed the specific amplification (Fig. S1). The efficiency of qPCR reaction varied from 92.89 for *28S rRNA* to 97.76 for *18S rRNA*, and correlation coefficient ranged from 0.992 (for *18S rRNA*) to 0.999 (for *β-TUB* and *UBQ*), respectively (Table 1).

### Expression profiles of candidate reference genes

**Figure 2:**
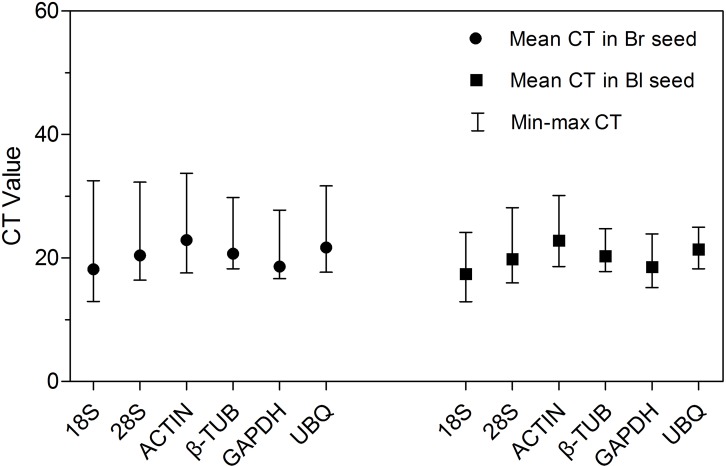
CT values of qRT-PCR for the six candidate reference genes. Expression level of each reference gene displayed as CT values in all treatments in brown and black seeds, respectively. The medium, maximum and minimum CT values of each sample were calculated. Four biological replicates with two technical replicates of each for all samples were applied. Bl, black; Br, brown.

Amplification of *ACTIN* in each cDNA sample of *S. aralocaspica* produced a single specific band with a predicted size (approximately 150 bp) (Fig. S2), which means the cDNA from the reverse transcription of *S. aralocaspica* total RNA was appropriate for the following analysis (Li et al., 2012; Wei et al., 2013). Detection of the expression level of all samples in both types of seeds revealed some variations amongst the six reference genes (Fig. 2; Table S2). The cycle threshold (CT, Bustin et al., 2009) value for the six genes ranged from 12.95 to 33.73, while the majority of these values were between 17 and 23 in all tested samples. *ACTIN* showed the highest mean CT value (22.92 and 22.84 in brown and black seeds, respectively), which represented a relatively lower expression level. By contrast, *18S rRNA* displayed high expression level compared to other reference genes in brown seed (CT = 18.20) and black seed (CT = 17.42). Among them, *β-TUB* and *UBQ* showed smaller gene expression variation (below 7 cycles) among studied reference genes in black seed, whereas *18S rRNA*, *28S rRNA* and *ACTIN* displayed much higher expression variation (more than 15 cycles; Table S2) among all studied genes in brown seed. The wide range variation in expression of the six tested reference genes suggests that each reference gene will not keep a constant expression level in different samples of *S. aralocaspica*, and selection of the reliable reference gene to normalize gene expression under certain condition is needed.

### Expression stability of candidate reference genes

Since the six candidate reference genes showed wide variation in expression level in different sample sets, it is necessary to use statistical method to rank the stability of these genes and determine the least reference gene number in normalization for accurate gene expression profiling under given conditions. In the present study, three most widely used algorithms, geNorm, NormFinder and BestKeeper were employed in the analysis.

#### GeNorm analysis

**Figure 3:**
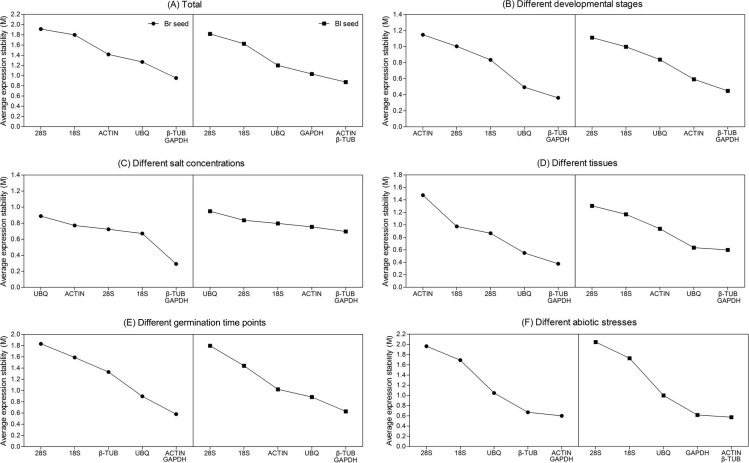
Gene expression stability and ranking of six reference genes based on geNorm algorithm. The cutoff *M* value is proposed to be 1.5, a lower *M* value indicates more stable expression. The least stable genes are on the left, and the most stable ones are on the right. (A) All tested samples; (B) different developmental stages; (C) different salt concentrations; (D) different seedling tissues (root, stem, and leaf); (E) different germination time points; (F) different abiotic stresses. Bl, black; Br, brown.

The expression stability value (*M*) for each reference gene is calculated based on the average pairwise variation value with other genes by geNorm algorithm. Stepwise exclusion of the least stable gene allows the genes to be ranked according to their *M* value. It is recommended that an *M* value below the threshold of 1.5 can be accepted. According to the geNorm algorithm, the lower the *M* value is, the higher the gene expression stability is. The results achieved with geNorm algorithm are showed in Fig. 3. For all the tested samples, *β-TUB* and *GAPDH* (for brown seed) or *ACTIN* and *β-TUB* (for black seed) were the two most stable genes, whereas *28S rRNA* was the least stable in both types of seeds (Fig. 3A). The *β-TUB* and *GAPDH* were proved to be the best candidates for normalization in two types of seeds at different developmental stages (Fig. 3B), salt treatments (Fig. 3C) and different tissues (Fig. 3D). However, *ACTIN* (for brown seed) and *28S rRNA* (for black seed) were shown to be the least stable genes at different developmental stages and tissues (Figs. 3B and 3D), while *UBQ* was the least stable gene under salt treatment (Fig. 3C). For different germination time points, *ACTIN* and *GAPDH* (for brown seed) or *β-TUB* and *GAPDH* (for black seed) were the most stable genes, while *28S rRNA* was the least stable (Fig. 3E). For stress treatment samples, *ACTIN* and *GAPDH* (for brown seed) or *ACTIN* and *β-TUB* (for black seed) were the two most stable genes, while *28S rRNA* was the least stable (Fig. 3F). Combining above analysis, *GAPDH* (for brown seed) and *β-TUB* (for black seed) were the most stable reference genes in six different sample sets.

**Figure 4:**
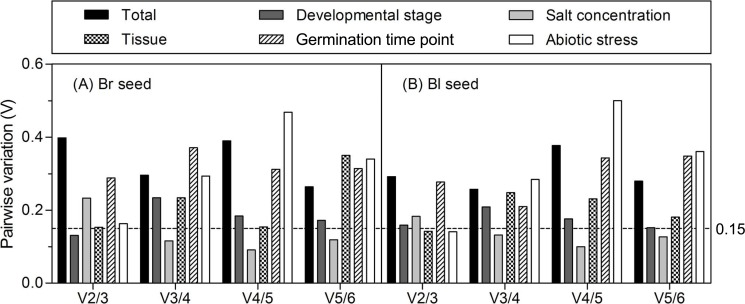
Pairwise variation (*V*) analyses of the candidate reference genes. The pairwise variation (*Vn*/*n* + 1) was analyzed for the normalization factors NF*n* and NF*n* + 1 by the geNorm program to determine the optimal number of reference genes for accurate normalization. The cutoff value was proposed to be 0.15, below which the inclusion of an additional reference gene is not necessary. Bl, black; Br, brown.

The optimal number of reference gene required for accurate normalization was calculated from the pairwise variation value (*Vn*/*n* + 1) to determine the effect of addition of another reference gene in normalization. A proposed cut-off value −0.15 is used to judge if it is necessary to include the *n* + 1 reference gene for normalization (Vandesompele et al., 2002). When *Vn*/*n* + 1 below 0.15, means that the addition of another reference has no significant effect in data normalization and is not necessary. Ideally, extra reference genes are included until the variation *Vn*/*n* + 1 drops below the given threshold. As shown in Fig. 4, when all the samples or samples from different germination time points were taken into account, all of the pairwise variations were higher than 0.15, suggesting that none of the combination was good enough for data normalization, much better reference genes should be included. For samples from the different developmental stages, the *V*2/3 was 0.131 in brown and 0.159 in black seed, respectively, which indicates that the third reference had no significant effect in both types of seeds and was not necessary to include. For different tissues, the *V*2/3 was 0.153 and 0.142 in brown and black seeds, respectively. Similar results were found for the different abiotic stresses with the *V*2/3 value slightly higher than 0.15 in brown seed (0.163) and lower in black seed (0.141), suggesting that two references were sufficient for normalizing data with different tissues and under abiotic stress conditions. For the *V*3/4 value below 0.15, three references were sufficient for normalizing gene expression in two types of seeds under salt stress condition.

#### NormFinder analysis

Different from geNorm, NormFinder program takes intra- and inter-group variation into account for normalization factor calculation. This algorithm ranks the set of candidate reference genes according to the stability of their expression patterns: a lower value means a good expression stability. The results of the Normfinder analysis of our data sets were summarized in Table 2. For all the samples pooled together, NormFinder identified *UBQ* (ranked 2nd and 3rd in brown and black seeds, respectively by geNorm) and *β-TUB* (ranked 1st in both types of seeds by geNorm) were the most stable genes, while *GAPDH* (ranked 1st and 2nd in brown and black seeds, respectively by geNorm) was the least stable gene in both types of seeds. For samples from different developmental stages and tissues, *β-TUB* and *GAPDH* were suggested to be the most stable genes in both types of seeds (similar to geNorm), while *ACTIN* and *28S rRNA* became the least stable genes with slight change in the ranking order compared to geNorm. For different salt concentration, NormFinder algorithm revealed that *β-TUB* and *GAPDH* (for brown seed) or *ACTIN* and *GAPDH* (for black seed) were the most stable genes, while *UBQ* was the least stable gene. These results were consistent with those obtained by geNorm, except for *β-TUB* in black seed (the rank changed from 1st in geNorm to 5th in NormFinder). For different germination time points, NormFinder analysis indicated that *ACTIN* and *GAPDH* (for brown seed) or *GAPDH* and *UBQ* (for black seed) were the most stable genes, while *28S rRNA* and *β-TUB* (ranked 1st and 3rd in black and brown seeds, respectively by geNorm) were the least stable genes in both types of seeds. For abiotic stress, *β-TUB* and *ACTIN* were the most stable genes in both types of seeds, while with a slight difference in ranking order compared to geNorm, such as *β-TUB* ranking from 2nd in geNorm to 1st in NormFinder in brown seed. Notably, both geNorm and NormFinder revealed that *18S rRNA* and *28S rRNA* were the least stable genes in both types of seeds. Taken together, results of these two algorithms suggest that *GAPDH* might be a quite stable reference gene in both types of seeds among different sample sets.

**Table 2:** Candidate genes ranked according to their expression stability value estimated by NormFinder.

Seed type	Rank	Total		Developmental stage		Salt concentration		Tissue		Germination time point		Abiotic stress	
Brown seed	1	*UBQ*	*0.361*	*β-TUB*	0.232	*β-TUB*	0.250	*β-TUB*	0.302	*ACTIN*	0.294	*β-TUB*	0.204
	2	*β-TUB*	*0.377*	*GAPDH*	0.294	*GAPDH*	0.269	*GAPDH*	0.344	*GAPDH*	0.453	*ACTIN*	0.380
	3	*ACTIN*	*0.467*	*UBQ*	0.379	*ACTIN*	0.381	*18S*	0.480	*18S*	0.546	*GAPDH*	0.432
	4	*28S*	*0.521*	*18S*	0.591	*28S*	0.389	*UBQ*	0.526	*UBQ*	0.686	*UBQ*	0.799
	5	*18S*	*0.598*	*ACTIN*	0.711	*18S*	0.389	*28S*	0.690	*28S*	0.729	*18S*	0.820
	6	*GAPDH*	*0.607*	*28S*	0.720	*UBQ*	0.540	*ACTIN*	1.296	*β-TUB*	1.097	*28S*	0.849
Black seed	1	*β-TUB*	0.348	*GAPDH*	0.260	*ACTIN*	0.253	*GAPDH*	0.258	*GAPDH*	0.430	*β-TUB*	0.325
	2	*UBQ*	0.407	*β-TUB*	0.423	*GAPDH*	0.306	*β-TUB*	0.295	*UBQ*	0.533	*ACTIN*	0.424
	3	*28S*	0.416	*UBQ*	0.477	*28S*	0.323	*UBQ*	0.327	*ACTIN*	0.535	*GAPDH*	0.432
	4	*18S*	0.436	*18S*	0.518	*18S*	0.341	*18S*	0.493	*18S*	0.611	*UBQ*	0.552
	5	*GAPDH*	0.446	*ACTIN*	0.519	*β-TUB*	0.359	*28S*	0.640	*β-TUB*	0.717	*28S*	0.813
	6	*ACTIN*	0.450	*28S*	0.640	*UBQ*	0.571	*ACTIN*	0.727	*28S*	0.856	*18S*	0.813

#### BestKeeper analysis

The BestKeeper index is based on the average CT value of each duplicated reaction. The variation in gene expression is calculated based on the standard deviation (SD) and coefficient of variance (CV). Genes with the lowest SD and the lowest CV values are the most stable. Any proposed reference gene with an SD ¿ 1 is considered to be inconsistent and should be excluded. In the present study, the ranking of the candidate genes are shown in Table 3. For samples from the total and different developmental stages, *β-TUB* and *GAPDH* (for brown seed) or *ACTIN* and *β-TUB* (for black seed) were the most stable genes; for salt treatment, *β-TUB* and *GAPDH* (for brown seed) or *ACTIN* and *GAPDH* (for black seed) were the most stable genes; for different tissues, *β-TUB* and *GAPDH* (for brown seed) or *UBQ* and *GAPDH* (for black seed) were the most stable genes; for different germination time points, *GAPDH* and *ACTIN* (for brown seed) or *UBQ* and *GAPDH* (for black seed) showed the most stable expression; for stress treatment samples, *GAPDH* and *ACTIN* (for brown seed) or *ACTIN* and *GAPDH* (for black seed) were the most stable genes. These results were largely consistent with those obtained from geNorm and NormFinder. However, due to the different statistical algorithms of three methods, there were some differences in the reference ranking order. It is notable that *28S rRNA* was the least stable gene in all three algorithms, except for the salt treatment sample (summarized in Table S3).

**Table 3:** Candidate genes ranking according to the expression stability value calculated by BestKeeper.

Seed type	Total			Developmental stage			Salt concentration			Tissue			Germination time point			Abiotic stress		
	Rank	SD	CV	Rank	SD	CV	Rank	SD	CV	Rank	SD	CV	Rank	SD	CV	Rank	SD	CV
Brown seed	*β-TUB*	0.70	3.67	*β-TUB*	0.47	2.38	*β-TUB*	0.28	1.47	*β-TUB*	0.54	2.73	*GAPDH*	0.55	2.91	*GAPDH*	0.44	2.39
	*GAPDH*	0.73	3.79	*GAPDH*	0.60	3.00	*GAPDH*	0.34	1.94	*GAPDH*	0.60	3.05	*ACTIN*	0.80	3.39	*ACTIN*	0.56	2.43
	*ACTIN*	0.94	4.19	*ACTIN*	0.66	3.33	*28S*	0.50	2.32	*UBQ*	0.92	4.38	*UBQ*	1.06	4.24	*β-TUB*	0.60	2.97
	*UBQ*	1.38	6.46	*UBQ*	0.79	3.84	*ACTIN*	0.51	2.68	*18S*	1.05	5.53	*β-TUB*	1.28	5.15	*UBQ*	1.18	5.28
	*18S*	1.96	10.05	*18S*	0.99	5.60	*18S*	0.58	3.61	*28S*	1.05	6.16	*18S*	2.05	9.96	*18S*	1.99	10.34
	*28S*	2.01	11.04	*28S*	1.04	6.45	*UBQ*	0.76	3.87	*ACTIN*	1.64	7.87	*28S*	2.61	11.89	*28S*	2.34	11.10
Black seed	*ACTIN*	0.87	3.80	*β-TUB*	0.36	1.77	*ACTIN*	0.36	1.63	*UBQ*	0.39	1.87	*UBQ*	0.41	1.91	*ACTIN*	0.70	3.04
	*β-TUB*	0.88	4.70	*ACTIN*	0.41	1.83	*GAPDH*	0.46	2.45	*GAPDH*	0.56	2.88	*GAPDH*	0.79	4.10	*GAPDH*	0.70	3.73
	*GAPDH*	0.95	4.73	*GAPDH*	0.45	2.52	*β-TUB*	0.65	3.28	*β-TUB*	0.58	3.04	*ACTIN*	0.98	4.12	*β-TUB*	0.86	4.11
	*UBQ*	1.04	4.85	*UBQ*	0.87	4.20	*28S*	0.67	3.70	*18S*	0.85	4.81	*β-TUB*	1.24	5.90	*UBQ*	0.91	4.14
	*28S*	1.65	9.35	*18S*	1.04	6.26	*18S*	0.69	3.95	*28S*	0.91	5.06	*18S*	1.75	9.40	*18S*	1.78	9.72
	*18S*	1.85	9.46	*28S*	1.16	6.57	*UBQ*	0.71	4.19	*ACTIN*	1.20	5.65	*28S*	2.46	11.58	*28S*	2.22	10.83

### Validation of the best performance reference genes

**Figure 5:**
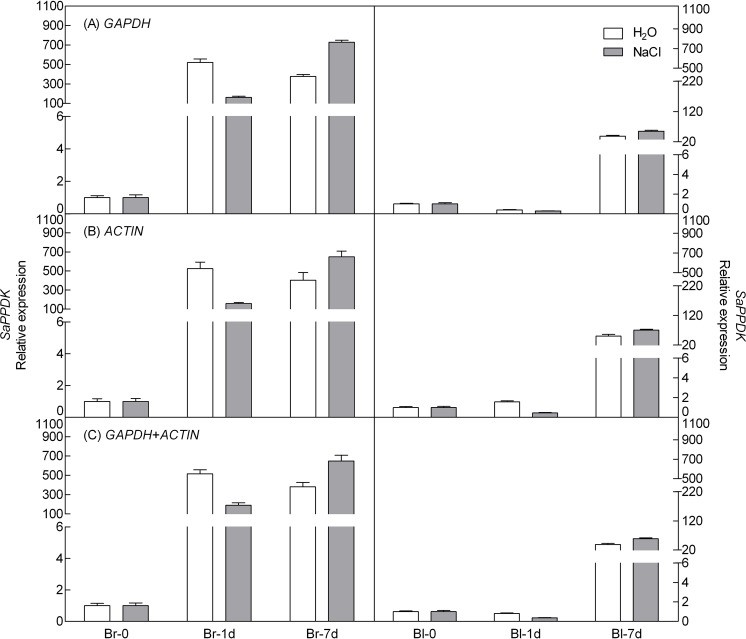
Relative quantification of *PPDK* expression using the selected reference gene(s). Relative expression of *PPDK* was normalized using the single most stable reference gene *GAPDH* (A), *ACTIN* (B) and their combination *GAPDH* + *ACTIN* (C) in sample sets under 300 mM NaCl treatment. Bl, black seed; Br, brown seed.

**Figure 6:**
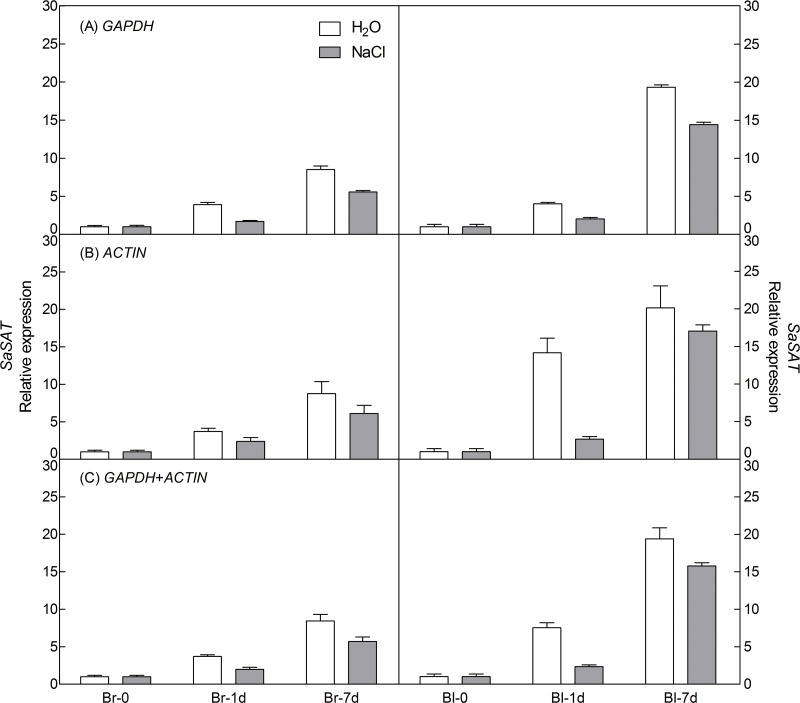
Relative quantification of *SAT* expression using the selected reference gene(s). Relative expression of *SAT* was normalized using the single most stable reference gene *GAPDH* (A), *ACTIN* (B) and their combination *GAPDH* + *ACTIN* (C) in sample sets under 300 mM NaCl treatment. Bl, black seed; Br, brown seed.

To demonstrate the reliability of our analysis with candidate reference genes, the relative expression level of a photosynthesis-related gene-pyruvate orthophosphate dikinase (*PPDK*) of C4 pathway and a salt tolerance-related gene-salt-induced AAA-Type ATPase (*SAT*) of *S. aralocaspica* were examined during germination under 300 mM salt treatment, using the most stable reference genes *GAPDH* (at germination time points), *ACTIN* (under abiotic stress) and their combination (*GAPDH* + *ACTIN*) for normalization (Figs. 5 and 6). The stability of these two reference genes were further confirmed by three algorithms when combined germination time point with abiotic stress together (Table S4). The analysis revealed that the transcript abundance of these two target genes increased significantly during germination in both types of seeds. The relative expression profile of each gene was much similar across the experimental sets when normalized using *GAPDH* or *ACTIN*, or *GAPDH* + *ACTIN*, although they showed a slight difference. However, as Figs. 5 and 6 showed, the relative transcript abundance for each target gene was dependent on the reference gene(s) used for normalization. When the expression of these two genes was normalized using a combination of *GAPDH* and *ACTIN*, which was identified as the most stable references by geNorm, the fold change was between those obtained by using either *GAPDH* or *ACTIN* as the single reference gene. This result clearly indicated that the utilization of more than one reference gene in normalization provided a more accurate representation of target gene expression when tested across variable experimental conditions and reinforced the importance of reference gene validation prior to experimental application.

## Discussion

*Suaeda aralocaspica* has evolved different mechanisms to cope with the harsh environments, e.g., single-cellular C4 photosynthesis pathway, seed heteromorphism, requirement of salinity for optimal growth, etc. (Voznesenskaya et al., 2001; Wang et al., 2008; Cao et al., 2015). However, the molecular mechanisms underpinning salt tolerance are not well understood. Transcriptional analysis with qRT-PCR is meaningful to reveal the function of stress tolerance-related genes, and which can highly improve the quantification of gene expression profiles (Fan et al., 2013; Yan et al., 2014). Previous studies have demonstrated that appropriate internal reference genes are the prerequisite to ensure accurate qRT-PCR analysis (Li et al., 2015), and different references should be employed for different species or in different experimental treatments (Løvdal & Lillo, 2009; Chen et al., 2011; Zhu et al., 2013). However, the direct transfer of traditional and proposed novel candidate reference genes to non-model plants are hampered by the limited availability of genomic sequences, e.g., the desert plant species in Amaranthaceae family. Therefore, in the present study, we analyzed a set of commonly used housekeeping genes in C4 halophyte *S. aralocaspica* for the normalization of gene expression analysis using qRT-PCR for the first time. Results indicate that *β-TUB*, *GAPDH* and *ACTIN* appeared to be the top three reference genes in most of the tested sample sets, while *18S* and *28S rRNA* were always ranked poorly and seemed to be unsuitable for using as internal control in *S. aralocaspica*.

In the present study, only few discrepancies were revealed among three analytical programs (Table S3), which might be caused by distinct algorithms and statistical procedures. geNorm selects the least variable genes with a low intra-group variation and approximately the same non-vanishing inter-group variation (Vandesompele et al., 2002). Bestkeeper selects the least variable gene using the geometric mean of the raw data (Pfaffl, Prgomet & Neuvians, 2004) and results of the whole six group samples were generally consistent with that of geNorm. In comparison, NormFinder does this with minimal combined inter- and intra-group expression variation (Andersen, Jensen & Orntoft, 2004), which can have a notable effect on the ranking of the subsequent gene stability (Exposito-Rodriguez et al., 2008). In the present study, the stability of *GAPDH* (for brown seed) or *ACTIN* (for black seed) was significantly dropped from the first place to the lowest position whereas *β-TUB* and *UBQ* ranked the top position by NormFinder algorithm compared to that of geNorm in the whole sample pools. Combining the brown and black seeds as a whole, the expression stability was further analyzed using above three methods (Table S5). The results were consistent with what have shown in Table S3 and revealed that, *β-TUB* can serve as the most reliable internal control for accurate normalization in all sample pools, especially by geNorm, which was recently suggested as one of the best methods to determine the reference gene in qRT-PCR analysis (Gutierrez et al., 2008). Despite of this, different experimental treatments in the present study revealed their own best reference genes. *β-TUB* and *GAPDH* ranked the best under different developmental stages and tissues, *GAPDH* was the one under different germination time points and salt stress conditions, and *ACTIN* was suitable for various stress treatments. Previous studies showed that *GAPDH* was unstable in different tissues or experimental conditions (Jain et al., 2006; Marino et al., 2008; Løvdal & Lillo, 2009), while other study in Arabidopsis revealed that *GAPDH* was ranked among the 100 most stably expressed genes, whereas *TUB6* and *ACT2* has never represented in the top 100 (Czechowski et al., 2005). These results suggest that there is no universal reference gene for all plant species. However, no matter how the rank of gene stability changed, *18S rRNA* and *28S rRNA* in the present study were always the least reliable genes based on three algorithms. It was found that the most unstable genes would almost remain the same in all sample sets (Exposito-Rodriguez et al., 2008; Artico et al., 2010; Lin & Lai, 2010; Wan et al., 2010).

Multi-factor should be considered in decision of the most stable gene number, such as time, resources and accuracy requirements (Die et al., 2010). In the present study, the pairwise variations (*Vn*/*n* + 1) were assessed by geNorm algorithm to determine the optimal control gene number in different experiment series (cut-off value around 0.15), which indicated that only salt-stressed samples need a third gene to normalize gene expression, i.e., adding *18S rRNA* (for brown seed) or *ACTIN* (for black seed) in combination with *β-TUB* and *GAPDH* to calculate a normalization factor. However, it has also been suggested that this cut-off value is too strict (Vandesompele et al., 2002). So, in our work, although the values of *Vn*/*n* + 1 were slightly more than 0.15 in different developmental stages (V2/3 = 0.159 for black seed), tissues (V2/3 = 0.153 for brown seed) and abiotic stresses (V2/3 = 0.163 for brown seed), they were still staying within an acceptable range for heterogeneous sample sets (Hellemans et al., 2007; Chen et al., 2015). In addition, the inclusion of a third gene is not in correspondence with a decrease of the *V* value (Fig. 4). Thus, two best-scored genes should be sufficient for the normalization of gene expression in this case.

Salinity alters a wide range of metabolic processes in growing plant, and halophyte can acclimate to high salinity by modulating the levels of a number of polypeptides and mRNAs via profound changes in gene expression (Hurkman, 1992; Kosová et al., 2011). In the present study, two genes screened from our previous transcriptomic data of *S. aralocaspica* were used in qRT-PCR validation of the reliability of candidate reference genes under salt treatment: pyruvate orthophosphate dikinase (*PPDK*), which is the key rate-limiting enzyme of the photosynthesis in C4 pathway in converting pyruvate to PEP (Edwards et al., 1985; Hýsková et al., 2014); salt-induced AAA-Type ATPase (*SAT*), which acts as molecular motors involved in diverse cellular functions including vesicle trafficking, proteasome-mediated protein degradation and chaperone-like activity (Hanson & Whiteheart, 2005). Results showed that the transcripts of *PPDK* were up-regulated under salt treatment after germination for 7 days with the potential stable reference gene *GAPDH* or *ACTIN*, or their combination to normalize the expression. Notably, the expression level of *PPDK* in brown seed increased more than 150-fold after germination for 1d (germinated seeds) compared to that of the dry seed, while that of black seed did not significantly changed at the first day (non-germinated), which was consistent with the germination performance of heteromorphic seeds (Wang et al., 2008). Previous studies indicated that the transcript of *PPDK* increased during salt stress in a facultative halophyte *Mesembryanthemum crystallinum* (Michalowski et al., 1989), and the activity of PPDK was increased under salinity treatment in maize (Omoto, Taniguchi & Miyake, 2012). In the present study, the expression of salt-induced gene-*SAT* was up-regulated under 300 mM NaCl treatment after germination for 7 days but was lower than that in distilled water, which means that during seed germination a large amount of genes’ expression might change profoundly to prepare for seedling growth, including above two target genes. However, salt stress would affect the seed germination process, although *SAT* should be induced by salt treatment normally, e.g., the expression level of *mcSKD1* gene (a homolog of the AAA-type ATPase family found in *M. crystallinum*) was increased under 200 mM or more NaCl treatment in cultured plant cells (Jou et al., 2004) and reduced via RNA interference which significantly decreased salt tolerance of Arabidopsis (Ho et al., 2010), the effect of salt stimulus may not be stronger than the trigger primed by seed germination. This tendency was in agreement with the germination behavior of *S. aralocaspica*, in which as salt concentration increasing from 0 to 600 mM NaCl, the germination rate of heteromorphic seeds decreased significantly (Wang et al., 2008). Nevertheless, when the least stable reference gene *18S* or *28S* RNA was used for normalization (Fig. S3), the transcript level of *SaPPDK* and *SaSAT* (at 7th day of germination) in both types of seeds was 50-fold or 30-fold higher, respectively than that of using the most stable reference genes, which may lead to misinterpretation of target gene expression level. Taken all together, our results confirmed that the most stable reference genes (*GAPDH* and *ACTIN*) identified in current study could be used for the normalization of gene expression under salt stress at different germination time points for both types of seeds of *S. aralocaspica*.

Seed heteromorphism is thought to be a bet-hedging strategy for plants to adapt to environmental stress (Venable, 1985; Weber, 2009). *S. aralocaspica* produces dimorphic seeds with disparate forms and different germination characteristics: after imbibition for 8 h (our observation), the brown seed can reach approximately 100% germination percentage (Song et al., 2012); while the black seed germinates after 24–72 h of imbibition (our observation) and only to a very low percentage (Wang et al., 2008). In our previous study, the difference of transcriptional profiles between dimorphic seeds in germination process was revealed (J Cao et al., 2016, unpublished data). The results indicated that the expression of a large proportion of genes changed significantly at 3 h in brown seed whereas 8 h in black seed after imbibition, which means that a series of physiological and biochemical events in germination may take place earlier in brown seed than that in black seed, and the transcriptional change was much greater in brown seed than that of black seed. Among the differential expressed transcript-derived fragments (TDF) identified with known sequences in the database, two commonly used reference genes, *GAPDH* and translation initiation factor (*eIF-4α*) were also detected in both types of seeds of *S. aralocaspica* (J Cao et al., 2016, unpublished data), which suggests that not only the germination-related genes but also the housekeeping genes may display different transcriptional profiles between the brown and black seeds. Consistently, in current study, the three most suitable reference genes showed different performance in two types of seeds under various conditions (Table S3). However, the different characteristics shown in germination between dimorphic seeds of *S. aralocaspica* will not be transferred to the descendants, and which will soon disappear in later seedling stage and present no significant difference in growth and physiological responses in the descendants with or without salinity (Cao et al., 2015).

## Supplemental Information

10.7717/peerj.1697/supp-1 Table S1 Gene description and nucleotide sequence identity with homolog species of six reference gene candidates from BLAST in NCBI

10.7717/peerj.1697/supp-2 Table S2 CT values of six candidate reference genes of *S. aralocaspica* among 96 tested samples

10.7717/peerj.1697/supp-3 Table S3 Ranking of the candidate reference genes according to the stability value using geNorm, NormFinder and BestKeeper analyses Notes: 1 represents the most stable gene and 6 represents the least stable gene; G, geNorm; N, NormFinder; B, Bestkeeper.

10.7717/peerj.1697/supp-4 Table S4 Ranking of the candidate reference genes when combined germination time point with abiotic stress according to the stability value using geNorm, NormFinder and BestKeeper analyses

10.7717/peerj.1697/supp-5 Table S5 Ranking of the candidate reference genes according to the stability value using geNorm, NormFinder and BestKeeper analyses when combining the black and brown seed as a whole

10.7717/peerj.1697/supp-6 Figure S1 Dissociation curves for six candidate reference genes and two target genes, showing single peaks (A–H) in different colours of each figure represents performances of eight different rows in a 96-well plate.

10.7717/peerj.1697/supp-7 Figure S2 Internal quality control of cDNA synthesis The mixture of equal amount of cDNAs from all tested samples was used as the template. 1.0% agarose gel electrophoresis visualized the specific amplification for *ACTIN* gene with the expected size. 1–12 in different lanes represented the amplification of partial tested cDNA samples, the rest cDNA samples showed the same single specific band of *ACTIN* gene. M represented DNA size marker.

10.7717/peerj.1697/supp-8 Figure S3 Relative quantification of *PPDK* and *SAT* expression using the least stable reference gene Relative expression of these two target genes was normalized using the single least stable reference gene *18S rRNA* (A) and *28S rRNA* (B) in sample sets under 300 mM NaCl treatment.

10.7717/peerj.1697/supp-9 Data S1 The partial sequences of the six candidate reference genes According to the published sequences in Amaranthaceae or Caryophyllales, the partial sequences of the six candidate reference genes were obtained from homology cloning. These sequences have not been added to the GenBank database.

10.7717/peerj.1697/supp-10 Data S2 The raw data of qRT-PCR

## Additional Information and Declarations

### Data Availability

The following information was supplied regarding data availability: We have uploaded the raw data as Data S1 and  S2.
