# Supplementary material for: Validation of reference genes for quantitative RT-PCR normalization in Suaeda aralocaspica, an annual halophyte with heteromorphism and C4 pathway without Kranz anatomy
Source: PeerJ. 2016 Feb 11;4:e1697. doi: 10.7717/peerj.1697 (PMC4756755; doi:10.7717/peerj.1697)
Supplement: Table S1 [file peerj-04-1697-s001.docx]

**Table S1. Gene description and nucleotide sequence identity with homolog species of six reference gene candidates from BLAST in NCBI.**

| Gene symbol | Gene annotation | Function | Homolog species (Accession) | E-value | Identity (%) |
| --- | --- | --- | --- | --- | --- |
| *18S* | 18S ribosomal RNA gene | Cytosolic small ribosomal subunit | *Beta vulgaris* (FJ669720.1) | 5e-130 | 100 |
| *28S* | 18S ribosomal RNA gene | Cytosolic large ribosomal subunit | *Frankenia pulverulenta* ([HQ843448.1](http://www.ncbi.nlm.nih.gov/nucleotide/345287587?report=genbank&log$=nucltop&blast_rank=10&RID=WVV48XZP01R)) | 2e-69 | 99 |
| *ACTIN* | Beta-actin | Cytoskeletal structure protein | *Celosia argentea* ([HQ844002.1](http://www.ncbi.nlm.nih.gov/nucleotide/322422112?report=genbank&log$=nucltop&blast_rank=1&RID=WVVR140A014)) | 5e-53 | 94 |
| *β-TUB* | Beta-tubulin | Cytoskeletal structure protein | *Spinacia oleracea* ([EF407952.1](http://www.ncbi.nlm.nih.gov/nucleotide/125662828?report=genbank&log$=nucltop&blast_rank=8&RID=WVVVKTV901R)) | 2e-54 | 91 |
| *GAPDH* | Glyceraldehyde-3-phosphate dehydrogenase | Glycolysis | [*Atriplex nummularia*](http://blast.ncbi.nlm.nih.gov/Blast.cgi#alnHdr_409574) ([U02886.1](http://www.ncbi.nlm.nih.gov/nucleotide/409574?report=genbank&log$=nucltop&blast_rank=3&RID=WVW1SXWA015)) | 0.0 | 88 |
| *UBQ* | ubiquitin protein10 | Protein degradation | [*Theobroma cacao*](http://blast.ncbi.nlm.nih.gov/Blast.cgi#alnHdr_590607612) ([XM_007020971.1](http://www.ncbi.nlm.nih.gov/nucleotide/590607612?report=genbank&log$=nucltop&blast_rank=10&RID=WVWBRTCX014)) | 1e-34 | 84 |
