# Supplementary material for: Validation of reference genes for quantitative RT-PCR normalization in Suaeda aralocaspica, an annual halophyte with heteromorphism and C4 pathway without Kranz anatomy
Source: PeerJ. 2016 Feb 11;4:e1697. doi: 10.7717/peerj.1697 (PMC4756755; doi:10.7717/peerj.1697)
Supplement: Table S2 [file peerj-04-1697-s002.docx]

**Table S2. CT values of six candidate reference genes of *S. aralocaspica* among 96 tested samples.**

| Seed type | Gene | Mean | Min | Max | Mean-min | Max-mean | Max-min |
| --- | --- | --- | --- | --- | --- | --- | --- |
| Brown seed | *18S* | 18.20 | 12.95 | 32.54 | 5.25 | 14.34 | 19.59 |
|  | *28S* | 20.42 | 16.40 | 32.29 | 4.02 | 11.87 | 15.89 |
|  | *ACTIN* | 22.92 | 17.56 | 33.73 | 5.35 | 10.81 | 16.17 |
|  | *β-TUB* | 20.71 | 18.26 | 29.80 | 2.45 | 9.09 | 11.54 |
|  | *GAPDH* | 18.64 | 16.65 | 27.75 | 1.98 | 9.11 | 11.10 |
|  | *UBQ* | 21.69 | 17.68 | 31.68 | 4.01 | 9.99 | 14.00 |
| Black seed | *18S* | 17.42 | 12.91 | 24.16 | 4.51 | 6.73 | 11.24 |
|  | *28S* | 19.83 | 15.98 | 28.14 | 3.85 | 8.32 | 12.17 |
|  | *ACTIN* | 22.84 | 18.60 | 30.12 | 4.23 | 7.29 | 11.52 |
|  | *β-TUB* | 20.30 | 17.82 | 24.75 | 2.48 | 4.45 | 6.93 |
|  | *GAPDH* | 18.54 | 15.21 | 23.91 | 3.33 | 5.37 | 8.70 |
|  | *UBQ* | 21.37 | 18.25 | 24.98 | 3.13 | 3.61 | 6.74 |
