# Supplementary material for: Validation of reference genes for quantitative RT-PCR normalization in Suaeda aralocaspica, an annual halophyte with heteromorphism and C4 pathway without Kranz anatomy
Source: PeerJ. 2016 Feb 11;4:e1697. doi: 10.7717/peerj.1697 (PMC4756755; doi:10.7717/peerj.1697)
Supplement: Table S3 — Notes: 1 represents the most stable gene and 6 represents the least stable gene; G, geNorm; N, NormFinder; B, Bestkeeper. [file peerj-04-1697-s003.docx]

**Table S3. Ranking of the candidate reference genes according to the stability value using geNorm, NormFinder and BestKeeper analyses**

|  | Brown seed | | | | Black seed | | | |
| --- | --- | --- | --- | --- | --- | --- | --- | --- |
|  | Gene | G | N | B | Gene | G | N | B |
| Total | *β-TUB* | 1 | 2 | 1 | *ACTIN* | 1 | 6 | 1 |
|  | *GAPDH* | 1 | 6 | 2 | *β-TUB* | 1 | 1 | 2 |
|  | *UBQ* | 2 | 1 | 4 | *GAPDH* | 2 | 5 | 3 |
|  | *ACTIN* | 3 | 3 | 3 | *UBQ* | 3 | 2 | 4 |
|  | *18S* | 4 | 5 | 5 | *18S* | 4 | 4 | 6 |
|  | *28S* | 5 | 4 | 6 | *28S* | 5 | 3 | 5 |
| Developmental stage | *β-TUB* | 1 | 1 | 1 | *β-TUB* | 1 | 2 | 1 |
|  | *GAPDH* | 1 | 2 | 2 | *GAPDH* | 1 | 1 | 3 |
|  | *UBQ* | 2 | 3 | 4 | *ACTIN* | 2 | 5 | 2 |
|  | *18S* | 3 | 4 | 5 | *UBQ* | 3 | 3 | 4 |
|  | *28S* | 4 | 6 | 6 | *18S* | 4 | 4 | 5 |
|  | *ACTIN* | 5 | 5 | 3 | *28S* | 5 | 6 | 6 |
| Salt concentration | *β-TUB* | 1 | 1 | 1 | *β-TUB* | 1 | 5 | 3 |
|  | *GAPDH* | 1 | 2 | 2 | *GAPDH* | 1 | 2 | 2 |
|  | *18S* | 2 | 5 | 5 | *ACTIN* | 2 | 1 | 1 |
|  | *28S* | 3 | 4 | 3 | *18S* | 3 | 4 | 5 |
|  | *ACTIN* | 4 | 3 | 4 | *28S* | 4 | 3 | 4 |
|  | *UBQ* | 5 | 6 | 6 | *UBQ* | 5 | 6 | 6 |
| Tissue | *β-TUB* | 1 | 1 | 1 | *β-TUB* | 1 | 2 | 3 |
|  | *GAPDH* | 1 | 2 | 2 | *GAPDH* | 1 | 1 | 2 |
|  | *UBQ* | 2 | 4 | 3 | *UBQ* | 2 | 3 | 1 |
|  | *28S* | 3 | 5 | 5 | *ACTIN* | 3 | 6 | 6 |
|  | *18S* | 4 | 3 | 4 | *18S* | 4 | 4 | 4 |
|  | *ACTIN* | 5 | 6 | 6 | *28S* | 5 | 5 | 5 |
| Germination time point | *ACTIN* | 1 | 1 | 2 | *β-TUB* | 1 | 5 | 4 |
|  | *GAPDH* | 1 | 2 | 1 | *GAPDH* | 1 | 1 | 2 |
|  | *UBQ* | 2 | 4 | 3 | *UBQ* | 2 | 2 | 1 |
|  | *β-TUB* | 3 | 6 | 4 | *ACTIN* | 3 | 3 | 3 |
|  | *18S* | 4 | 3 | 5 | *18S* | 4 | 4 | 5 |
|  | *28S* | 5 | 5 | 6 | *28S* | 5 | 6 | 6 |
| Abiotic stress | *ACTIN* | 1 | 2 | 2 | *ACTIN* | 1 | 2 | 1 |
|  | *GAPDH* | 1 | 3 | 1 | *β-TUB* | 1 | 1 | 3 |
|  | *β-TUB* | 2 | 1 | 3 | *GAPDH* | 2 | 3 | 2 |
|  | *UBQ* | 3 | 4 | 4 | *UBQ* | 3 | 4 | 4 |
|  | *18S* | 4 | 5 | 5 | *18S* | 4 | 5 | 5 |
|  | *28S* | 5 | 6 | 6 | *28S* | 5 | 6 | 6 |

Notes: 1 represents the most stable gene and 6 represents the least stable gene; G: geNorm, N: NormFinder, B: Bestkeeper.
