# Supplementary material for: Validation of reference genes for quantitative RT-PCR normalization in Suaeda aralocaspica, an annual halophyte with heteromorphism and C4 pathway without Kranz anatomy
Source: PeerJ. 2016 Feb 11;4:e1697. doi: 10.7717/peerj.1697 (PMC4756755; doi:10.7717/peerj.1697)
Supplement: Table S4 [file peerj-04-1697-s004.docx]

**Table S4. Ranking of the candidate reference genes when combined germination time point with abiotic stress according to the stability value using geNorm, NormFinder and BestKeeper analyses**

| Seed type | Rank |  | geNorm |  | NormFinder |  | BestKeeper | |
| --- | --- | --- | --- | --- | --- | --- | --- | --- |
|  |  |  |  |  |  |  | SD | CV |
| Brown seed | 1 | *ACTIN* | 0.594 | *ACTIN* | 0.283 | *GAPDH* | 0.85 | 5.07 |
|  | 2 | *GAPDH* | 0.594 | *GAPDH* | 0.333 | *ACTIN* | 0.99 | 4.61 |
|  | 3 | *β-TUB* | 0.940 | *β-TUB* | 0.607 | *β-TUB* | 1.21 | 5.87 |
|  | 4 | *UBQ* | 1.229 | *UBQ* | 0.614 | *UBQ* | 1.35 | 5.96 |
|  | 5 | *18S* | 1.890 | *28S* | 0.627 | *18S* | 2.51 | 12.7 |
|  | 6 | *28S* | 2.140 | *18S* | 0.643 | *28S* | 2.84 | 13.06 |
| Black seed | 1 | *β-TUB* | 0.679 | *ACTIN* | 0.396 | *GAPDH* | 0.82 | 4.36 |
|  | 2 | *GAPDH* | 0.679 | *GAPDH* | 0.454 | *ACTIN* | 0.88 | 3.84 |
|  | 3 | *ACTIN* | 0.733 | *β-TUB* | 0.458 | *UBQ* | 0.90 | 3.99 |
|  | 4 | *UBQ* | 1.068 | *UBQ* | 0.624 | *β-TUB* | 0.99 | 4.72 |
|  | 5 | *18S* | 1.706 | *18S* | 0.683 | *18S* | 1.85 | 10.02 |
|  | 6 | *28S* | 2.021 | *28S* | 0.815 | *28S* | 2.41 | 11.66 |
