# Supplementary material for: Validation of reference genes for quantitative RT-PCR normalization in Suaeda aralocaspica, an annual halophyte with heteromorphism and C4 pathway without Kranz anatomy
Source: PeerJ. 2016 Feb 11;4:e1697. doi: 10.7717/peerj.1697 (PMC4756755; doi:10.7717/peerj.1697)
Supplement: Table S5 [file peerj-04-1697-s005.docx]

**Table S5. Ranking of the candidate reference genes according to the stability value using geNorm, NormFinder and BestKeeper analyses when combining the black and brown seed as a whole**

| Seed type | Rank |  | geNorm |  | NormFinder |  | BestKeeper | |
| --- | --- | --- | --- | --- | --- | --- | --- | --- |
|  |  |  |  |  |  |  | SD | CV |
| Total | 1 | *β-TUB* | 0.978 | *β-TUB* | 0.324 | *GAPDH* | 0.88 | 4.69 |
|  | 2 | *GAPDH* | 0.978 | *UBQ* | 0.353 | *β-TUB* | 0.99 | 4.32 |
|  | 3 | *ACTIN* | 1.177 | *ACTIN* | 0.421 | *ACTIN* | 1.05 | 5.13 |
|  | 4 | *UBQ* | 1.340 | *GAPDH* | 0.429 | *UBQ* | 1.26 | 5.85 |
|  | 5 | *18S* | 1.735 | *28S* | 0.466 | *28S* | 1.69 | 8.39 |
|  | 6 | *28S* | 1.883 | *18S* | 0.477 | *18S* | 1.75 | 9.80 |
| Developmental stage | 1 | *β-TUB* | 0.467 | *GAPDH* | 0.255 | *β-TUB* | 0.45 | 2.26 |
|  | 2 | *GAPDH* | 0.467 | *β-TUB* | 0.323 | *GAPDH* | 0.52 | 2.91 |
|  | 3 | *UBQ* | 0.718 | *UBQ* | 0.394 | *ACTIN* | 0.61 | 2.75 |
|  | 4 | *ACTIN* | 0.858 | *ACTIN* | 0.491 | *UBQ* | 0.85 | 4.11 |
|  | 5 | *18S* | 1.034 | *18S* | 0.594 | *18S* | 1.02 | 6.56 |
|  | 6 | *28S* | 1.144 | *28S* | 0.648 | *28S* | 1.10 | 5.93 |
| Salt concentration | 1 | *β-TUB* | 0.534 | *ACTIN* | 0.245 | *ACTIN* | 0.45 | 2.04 |
|  | 2 | *GAPDH* | 0.534 | *GAPDH* | 0.253 | *GAPDH* | 0.48 | 2.60 |
|  | 3 | *ACTIN* | 0.718 | *β-TUB* | 0.255 | *β-TUB* | 0.50 | 2.59 |
|  | 4 | *18S* | 0.780 | *28S* | 0.290 | *28S* | 0.52 | 2.96 |
|  | 5 | *28S* | 0.813 | *18S* | 0.320 | *18S* | 0.62 | 3.86 |
|  | 6 | *UBQ* | 0.924 | *UBQ* | 0.526 | *UBQ* | 0.71 | 3.59 |
| Tissues | 1 | *β-TUB* | 0.559 | *GAPDH* | 0.201 | *GAPDH* | 0.58 | 2.95 |
|  | 2 | *GAPDH* | 0.559 | *β-TUB* | 0.243 | *β-TUB* | 0.62 | 3.20 |
|  | 3 | *UBQ* | 0.633 | *UBQ* | 0.397 | *UBQ* | 0.66 | 3.12 |
|  | 4 | *28S* | 0.974 | *18S* | 0.416 | *18S* | 0.96 | 5.66 |
|  | 5 | *18S* | 1.101 | *28S* | 0.624 | *28S* | 0.99 | 5.21 |
|  | 6 | *ACTIN* | 1.425 | *ACTIN* | 0.995 | *ACTIN* | 1.45 | 6.90 |
| Germination time point | 1 | *ACTIN* | 0.777 | *ACTIN* | 0.312 | *GAPDH* | 0.98 | 5.08 |
|  | 2 | *GAPDH* | 0.777 | *GAPDH* | 0.317 | *UBQ* | 1.02 | 4.59 |
|  | 3 | *UBQ* | 1.046 | *UBQ* | 0.414 | *ACTIN* | 1.18 | 4.91 |
|  | 4 | *β-TUB* | 1.250 | *β-TUB* | 0.469 | *β-TUB* | 1.77 | 8.32 |
|  | 5 | *18S* | 1.571 | *18S* | 0.661 | *18S* | 2.09 | 10.81 |
|  | 6 | *28S* | 1.851 | *28S* | 0.832 | *28S* | 2.68 | 12.23 |
| Abiotic stress | 1 | *ACTIN* | 0.636 | *β-TUB* | 0.245 | *GAPDH* | 0.97 | 5.15 |
|  | 2 | *GAPDH* | 0.636 | *ACTIN* | 0.306 | *ACTIN* | 1.02 | 4.32 |
|  | 3 | *β-TUB* | 0.746 | *GAPDH* | 0.326 | *β-TUB* | 1.06 | 5.09 |
|  | 4 | *UBQ* | 1.110 | *UBQ* | 0.552 | *UBQ* | 1.26 | 5.62 |
|  | 5 | *18S* | 1.861 | *28S* | 0.643 | *18S* | 2.29 | 11.98 |
|  | 6 | *28S* | 2.152 | *18S* | 0.645 | *28S* | 2.53 | 12.01 |
