# Supplementary material for: Validation of reference genes for quantitative RT-PCR normalization in Suaeda aralocaspica, an annual halophyte with heteromorphism and C4 pathway without Kranz anatomy
Source: PeerJ. 2016 Feb 11;4:e1697. doi: 10.7717/peerj.1697 (PMC4756755; doi:10.7717/peerj.1697)
Supplement: Figure S1 — (A–H) in different colours of each figure represents performances of eight different rows in a 96-well plate. [file peerj-04-1697-s006.docx]

**Supplemental Fig. S1** Dissociation curves for six candidate reference genes and two target genes, showing single peaks. A-H in different colours of each figure represents performances of eight different rows in a 96-well plate.


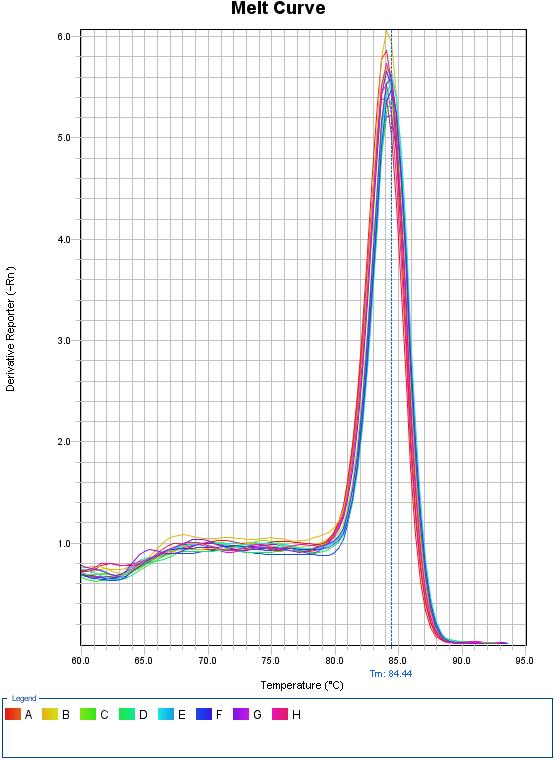


***18S***


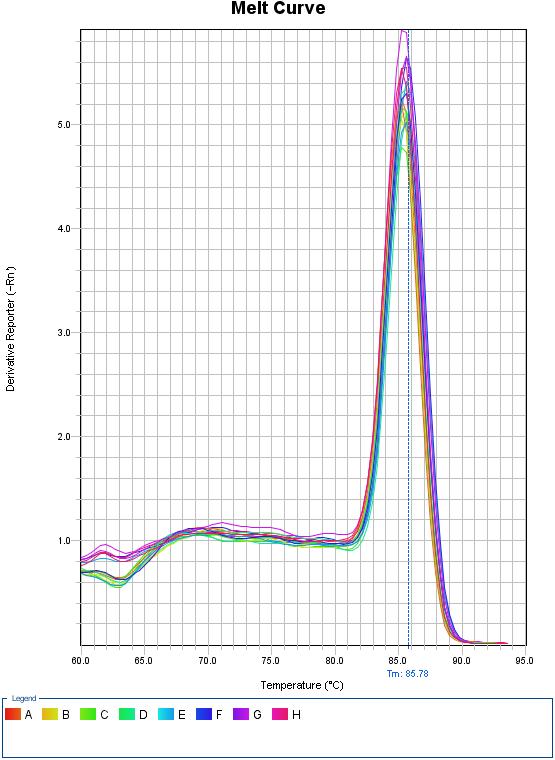


***28S***


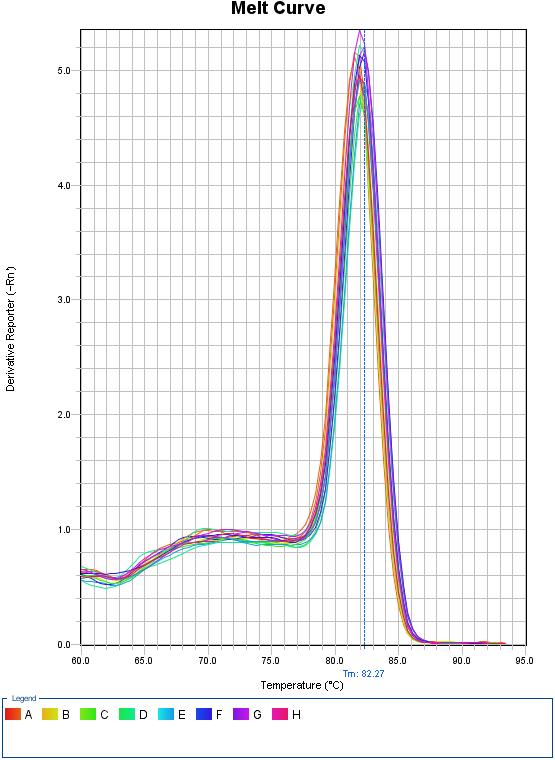


***ACTIN***


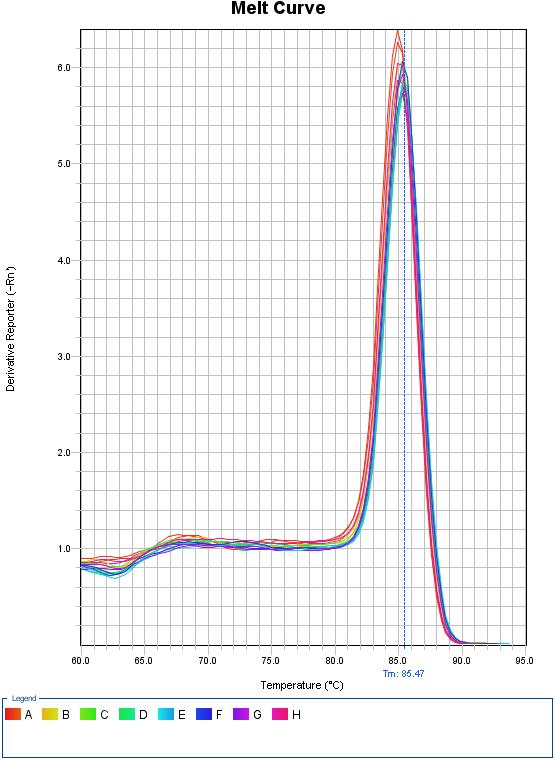


***β-TUB***


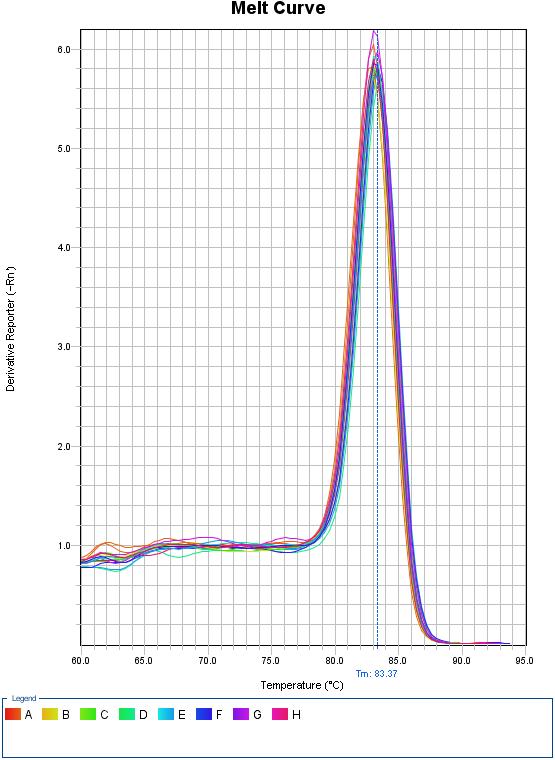


***GAPDH***


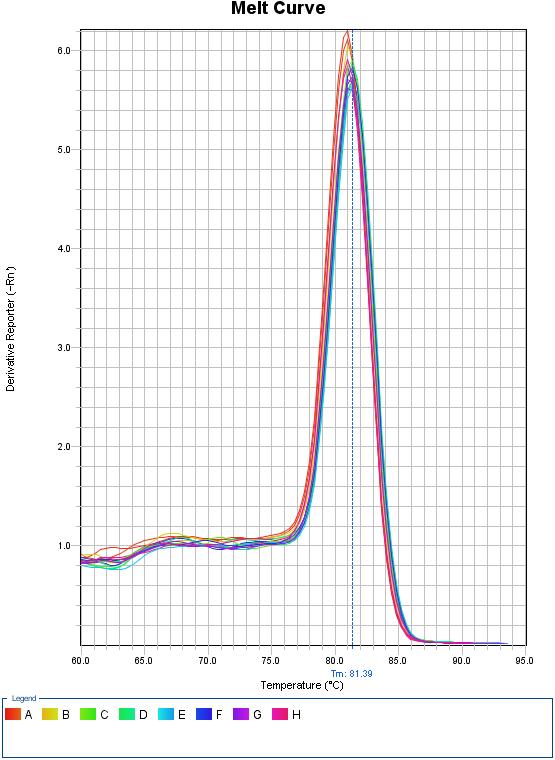


***UBQ***


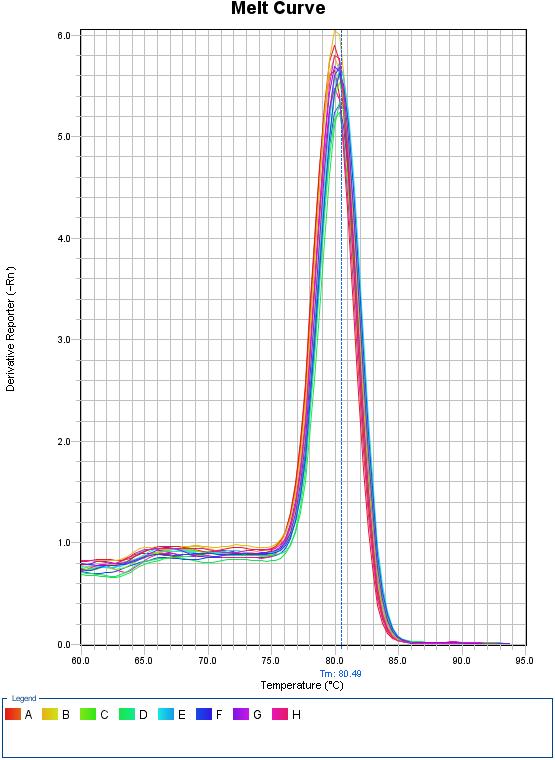


***PPDK***


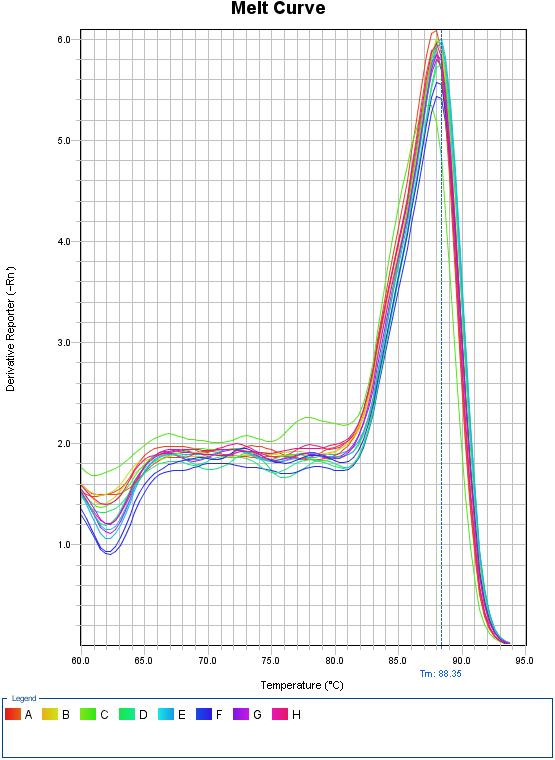


***SAT***

**Supplemental Fig. S1**
