# Supplementary material for: Validation of reference genes for quantitative RT-PCR normalization in Suaeda aralocaspica, an annual halophyte with heteromorphism and C4 pathway without Kranz anatomy
Source: PeerJ. 2016 Feb 11;4:e1697. doi: 10.7717/peerj.1697 (PMC4756755; doi:10.7717/peerj.1697)

**Supplemental Fig. S2** Internal quality control of cDNA synthesis.

The mixture of equal amount of cDNAs from all tested samples was used as the template. 1.0% agarose gel electrophoresis visualized the specific amplification for *ACTIN* gene with the expected size. 1-12 in different lanes represented the amplification of partial cDNA samples, the rest cDNA samples showed the same single specific band of *ACTIN* gene. M stand for DNA size marker.


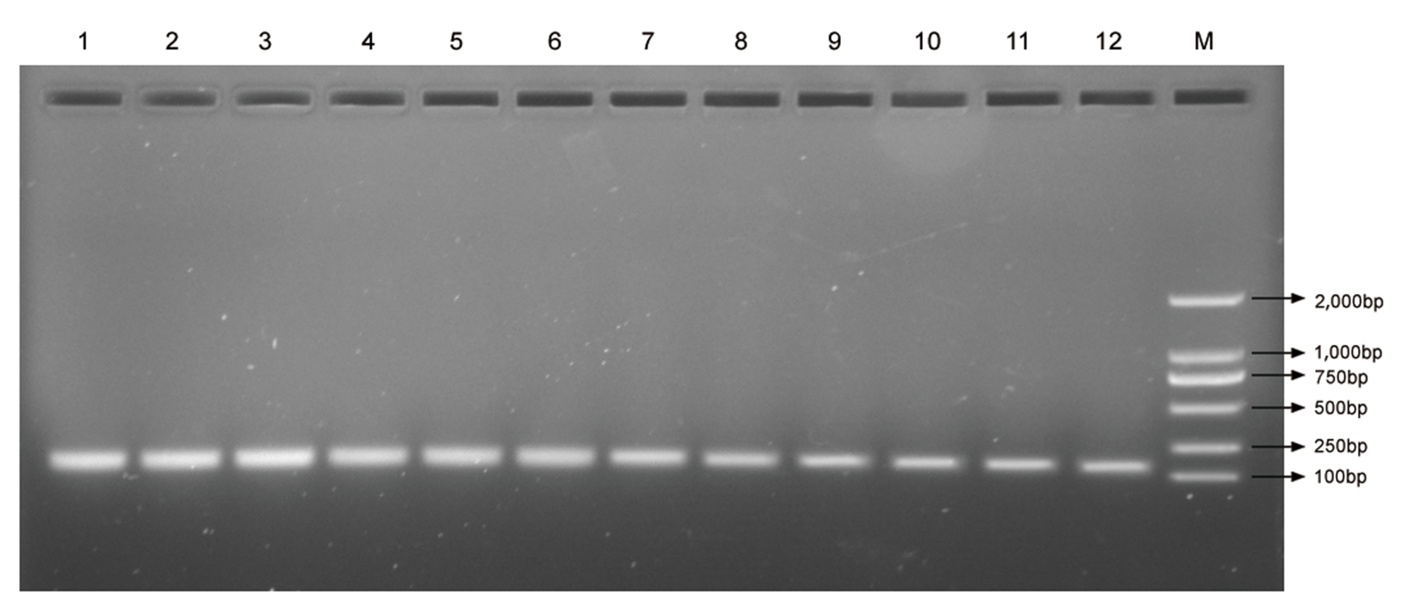

Supplement: Figure S2 — The mixture of equal amount of cDNAs from all tested samples was used as the template. 1.0% agarose gel electrophoresis visualized the specific amplification for ACTIN gene with the expected size. 1–12 in different lanes represented the amplification of partial tested cDNA samples, the rest cDNA samples showed the same single specific band of ACTIN gene. M represented DNA size marker. [file peerj-04-1697-s007.docx]
