# Supplementary material for: Validation of reference genes for quantitative RT-PCR normalization in Suaeda aralocaspica, an annual halophyte with heteromorphism and C4 pathway without Kranz anatomy
Source: PeerJ. 2016 Feb 11;4:e1697. doi: 10.7717/peerj.1697 (PMC4756755; doi:10.7717/peerj.1697)

**Supplemental Fig. S3** Relative quantification of *PPDK* and *SAT* expression using the least stable reference gene. Relative expression of these two target genes was normalized using the single least stable reference gene *18S rRNA* (A) and *28S rRNA* (B) in sample sets under 300 mM NaCl treatment.
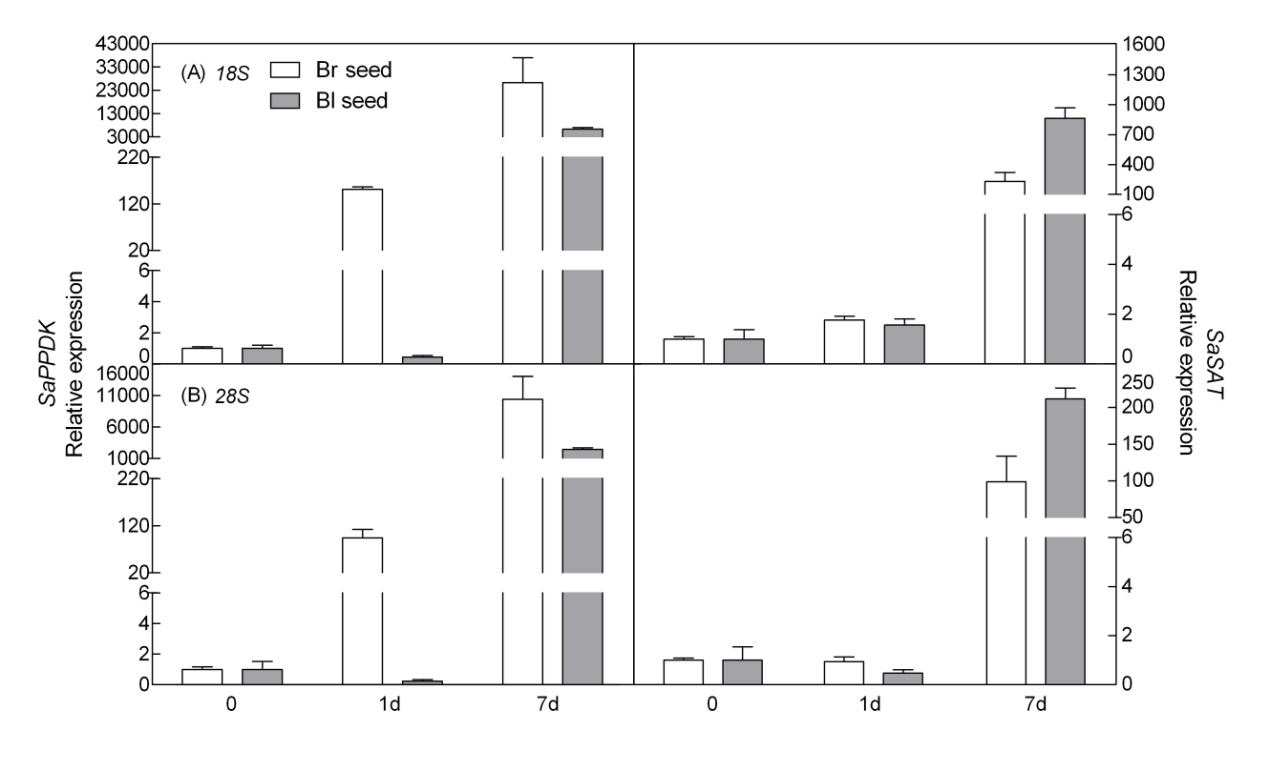

Supplement: Figure S3 — Relative expression of these two target genes was normalized using the single least stable reference gene 18S rRNA (A) and 28S rRNA (B) in sample sets under 300 mM NaCl treatment. [file peerj-04-1697-s008.docx]
