# Supplementary material for: Validation of reference genes for quantitative RT-PCR normalization in Suaeda aralocaspica, an annual halophyte with heteromorphism and C4 pathway without Kranz anatomy
Source: PeerJ. 2016 Feb 11;4:e1697. doi: 10.7717/peerj.1697 (PMC4756755; doi:10.7717/peerj.1697)
Supplement: Data S1 — According to the published sequences in Amaranthaceae or Caryophyllales, the partial sequences of the six candidate reference genes were obtained from homology cloning. These sequences have not been added to the GenBank database. [file peerj-04-1697-s009.docx]

According to the published sequences in Amaranthaceae or Caryophyllales, the partial sequences of the six candidate reference genes were obtained from homology cloning. These sequences have not been added to the GenBank database.

(1)*Sa18SrRNA*_1__(T150120-0216)RV-M_A77616_D06_1504063341J.seq (256bp)

GCGGCTTAATTTGACTCAACACGGGGAAACTTACCAGGTCCAGACATAGTAAGGATTGACAGACTGAGAGCTCTTTCTTGATTCTATGGGTGGTGGTGCATGGCCGTTCTTAGTTGGTGGAGCGATTTGTCTGGTTAATTCCGTTAACGAACGAGACCTCAGCCTGCTAACTAGCTATGCGGAGGTAGTCCTTCGCAGCTAGCTTCTTAGAGGGACTATGGCCTTTTAGGCCACGGAAGTTTGAGGCAATAACAGG

(2)*Sa28SrRNA*_4__(T150120-0216)RV-M_A77616_D04_1505062346J.seq (154bp)

GCCGACCCTGATCTTCTGTGAAGGGTTCGAGTGAGAGCATACCTGTCGGGACCCGAAAGATGGTGAACTATGCCTGAGCGGGGCGAAGCCAGAGGAAACTCTGGTGGAGGCCCGAAGCGATACTGACGTGCAAATCGTTC GTCTGACTTGGGTA

(3)*SaACTIN*_3__(T150120-0216)RV-M_A77616_H03_1505062342J.seq (149bp)

CCAAAGGCCAACAGAGAGAAGATGACCCAGATCATGTTTGAAACCTTCAATGTTCCAGCCATGTACGTTGCCATCCAGGCTGTTCTTTCTCTTTACGCTAGTGGTCGTACCACAGGTATTGTGCTTGATTCTGGTGATGGTGTGTCTCA

(4)*SaTUB*_2__(T150120-0216)RV-M_A77616_F05_1504092476J.seq (368bp)

GGACTCTCAAGCTAACTACACCAAGCTTTGGTGATTTGAACCATTTGATATCGGCCACCATGTCTGGAGTAACATGTTGTCTGAGGTTCCCAGGTCAATTAAACTCTGACCTTAGGAAACTTGCAGTCAACCTTATTCCATTCCCCAGGCTTCATTTCTTCATGGTGGGTTTCGCCCCACTAACATCTCGTGGGTCTCAGCAATACCGCTCCTTGACAGTCCCTGAGCTAACCCAGCAAATGTGGGATTCCAAGAACATGATGTGTGCCGCAGACCCTAGACATGGGCGTTACTTGACGGCTTCTGCCATGTTCCGAGGAAAGATGAGCACAAAGGAGGTTGATGAGCAGATGATCAACGTGCAGAAC

(5)*SaGAPDH*_10__(T150120-0216)RV-M_A77616_H05_1504092478J.seq (808bp)

GAGCTTGTTGCTGTTAACGATCCTTTCATCACCACTGACTACATGACATACATGTTTAAGTACGACAGTGTTCACGGTCAATGGAAACACCATGAGTTGAAGGTTAAGGATGAGAAGACTCTTCTTTTTGGTGACAAGCCAGTTACCGTATTCGGTTGCAGGAACCCAGAGGATATCCCATGGAGTTCGACTGGAGCTGACTTTGTTGTTGAGTCTACTGGTGTTTTCACTGACAAGGACAAGGCTGCTGCTCATTTGAAGGGTGGTGCCAAGAAGGTTGTCATATCTGCTCCTAGCAAGGATGCTCCCATGTTCGTCGTTGGTGTCAATGAGCATGAGTACAAGTCGGATCTTCACATTGTCTCCAATGCCAGTTGTACCACCAATTGCCTTGCTCCTTTGGCCAAGGTTATAAATGACAAGTTTGGCATCGTTGAGGGTCTTATGACCACTGTCCACTCCATCACTGCCACCCAAAAGACTGTTGACGGTCCATCAGCTAAGGACTGGCGAGGTGGAAGGGCTGCCTCATTTAACATCATTCCCAGCAGCACTGGAGCAGCTAAGGCTGTGGGAAAGGTTTTGCCTGCCCTCAATGGAAAATTGACTGGAATGGCTTTCCGTGTTCCAACTGTTGATGTTTCTGTTGTTGATCTTACTGTTAGACTTGAGAAGGCTGCTTCCTATGATCAGATTAAGGCTGCCATCAAGGAGGAGTCAGAGGGCAAGTTGAAGGGAGTTTTGGGTTACACCGACGAGGATGTTGTTTCCACTGATTTCATTGGTGACAACAGATCAAGTATTTTTG

(6)*SaUBQ*_A10__(T150120-0216)RV-M_A77616_D03_1505062338J.seq (162bp)

GCTAAGATTCAAGACAAGGAGGGTATCCCACCAGACCAGCAGAGGCTTATTTTTGCTGGGAAACAGCTAGAGGAAGGTAGAACACTGGCAGACTATAACATTCAGAAAGAATCAACCCTCCACCTGGTTCTTCGCCTTAGGGGAGGTATGCAGATTTTCGTG
